# Determination of the ED95 of intrathecal hyperbaric prilocaine with sufentanil for scheduled cesarean delivery: a dose-finding study based on the continual reassessment method

**DOI:** 10.1186/s12871-020-01199-0

**Published:** 2020-11-26

**Authors:** P. Goffard, Y. Vercruysse, R. Leloup, J-F Fils, S. Chevret, Y. Kapessidou

**Affiliations:** 1 Department of Anesthesiology, University Hospital Saint Pierre, Université Libre de Bruxelles, CHU Saint-Pierre, Rue Haute 322, 1000 Brussels, Belgium; 2 Ars Statistica S.P.R.L, Nivelles, Belgium; 3 Service de Biostatistique et Information Médicale, Hôpital Saint-Louis, Paris, France

**Keywords:** ED95, Hyperbaric prilocaine, Sufentanil, Cesarean section, Continual reassessment method

## Abstract

**Background:**

Scheduled cesarean section is routinely performed under spinal anesthesia using hyperbaric bupivacaine. The current study was undertaken to determine the clinically relevant 95% effective dose of intrathecal 2% hyperbaric prilocaine co-administered with sufentanil for scheduled cesarean section, using continual reassessment method.

**Methods:**

We conducted a dose-response, prospective, double-blinded study to determine the ED95 values of intrathecal hyperbaric prilocaine used with 2,5 mcg of sufentanil and 100 mcg of morphine for cesarean delivery. Each parturient enrolled in the study received an intrathecal dose of hyperbaric prilocaine determined by the CRM and the success or failure of the block was assessed as being the primary endpoint.

**Results:**

The doses given for each cohort varied from 35 to 50 mg of HP, according to the CRM, with a final ED95 lying between 45 and 50 mg of Prilocaine after completion of the 10 cohorts. Few side effects were reported and patients were globally satisfied.

**Conclusions:**

The ED95 of intrathecal hyperbaric prilocaine with sufentanil 2.5 μg and morphine 100 μg for elective cesarean delivery was found to be between 45 and 50 mg. It may be an interesting alternative to other long-lasting local anesthetics in this context.

**Trial registration:**

The study was registered on January 30, 2017 – retrospectively registered – and results posted at the public database clinicaltrials.gov (NCT03036384).

## Background

Scheduled cesarean section (CS) is routinely performed under spinal anesthesia using hyperbaric bupivacaine in combination with opioids [1–3]. Although efficient, its use is frequently associated with long-lasting motor block and adverse effects, mainly dose-dependent maternal hypotension [4, 5], increasing fetal risks [6, 7]. Considering the anesthetic efficacy, numerous studies have determined the dose-response relationship of the most commonly used intrathecal local anesthetics for caesarean section [8]. As well, it is currently admitted that the addition of intrathecal opioids enhances the potency of local anesthetics, while permitting a sparing effect [9, 10].

Nevertheless, nowadays, still remains the need to determine the “optimal” dose of local anesthetic for caesarean delivery, striking the balance between reliability and efficacy and adverse effects [11].

Hyperbaric prilocaine (HP) 2% is an intermediate-potency amide-type local anesthetic, providing short onset, intermediate duration of motor block and few side-effects [12, 13].

Several studies the last past years have shown its efficacy when applied for spinal anesthesia and have determined the appropriate doses for various ambulatory surgery procedures lasting up to 90 min [14–16].

First introduced for intrathecal use in 1965 [17], the former presentation of prilocaine was assessed in obstetrics for vaginal or cesarean delivery under continuous epidural anesthesia in 1968 [18, 19].

Good quality of anesthesia was reported with 1–2% formulations with no clinically relevant blood accumulation of prilocaine, although considerable doses had been administered via the continuous epidural mode [20].

Concerns regarding the stability of the solution related to production procedures [21] led to the withdrawal of prilocaine from the market in 1978, and no further investigations have been conducted in the obstetrics field ever since.

The new 2% intrathecal hyperbaric formulation commercialised in 2005, provides relevant advantages in terms of surgical anesthesia [22] and very low reported toxicity [23], thereby being an interesting alternative to long-lasting local anesthetics for cesarean section. Proposed doses for different surgical procedures vary largely, dictating the necessity for targeted studies.

The current study was undertaken to determine the clinically relevant 95% effective dose (ED95) of intrathecal 2% hyperbaric prilocaine co-administered with sufentanil for scheduled cesarean section. The doses were obtained using the continual reassessment method (CRM) [24], which has the advantage to estimate the targeted percentile on the dose-finding curve without extrapolation that lacks precision [25–27]. We also assessed clinical characteristics and side-effects profile associated with prilocaine’s doses used.

## Methods

### Design

We conducted a dose-response, prospective, double-blinded study to determine the ED95 values of intrathecal hyperbaric prilocaine used with 2,5 mcg of sufentanil and 100 mcg of morphine for cesarean delivery.

The study was approved by the institutional Medical Ethics Committee (President E. Stevens, Research Ethics Board number O.M.007; date of protocol approval 24 of March 2016; protocol number NB076201627436). It was retrospectively registered on January 30, 2017 and results posted at the public database clinicaltrials.gov (NCT03036384).

### Study population and setting

The present report was established according to ROBUST criteria for Bayesian based studies [28], SPIRIT statement for interventional trials [29] and CONSORT guidelines [30].

Healthy term parturients presenting to our hospital between 1st of April and 30th of November 2016 for elective cesarean delivery were enrolled in the study after signed written informed consent had been obtained.

Inclusion criteria were age between 18 and 40, American Society of Anesthesiologists physical status (ASA) class I-II, body weight less than 100 kg, height between 155 and 175 cm, singleton pregnancy, and gestational age of more than 37 completed weeks.

Exclusion criteria were active labor, ruptured membranes, three or more previous caesarean deliveries, diabetes or gestational diabetes, pregnancy induced hypertension or preeclampsia, intrauterine growth retardation, placenta praevia, congenital anomaly, standard contraindications to neuraxial block, neurological impairment, and known allergy to local anesthetics.

### Study protocol

All patients were premedicated with intravenous metoclopramide 10 mg, sodium citrate 30 ml and ranitidine 150 mg orally, 30 min before spinal anaesthesia. They received slowly upon arrival in the operating theatre 1000 ml of Ringer’s lactate solution via peripheral intravenous access as regular fluid therapy, which is standard care in our institution.

Continuous electrocardiography, pulse oximetry (SpO2) and non-invasive arterial blood pressure monitor were applied throughout the whole study protocol.

A combined spinal-epidural (CSE) was performed at the L3/L4 or L4/L5 interspace with the parturient in sitting position, under uterine and foetal heart rate monitoring.

Applying the midline approach, an 18G Tuohy needle (Vygon, Ecouen, France) was inserted into the epidural space using a loss-of-resistance-to-saline technique.

The spinal component was performed under aseptic conditions with a needle-through-needle technique using a 27G Whitacre needle (Vygon, Ecouen, France), with the orifice oriented cephalad.

Following observation of spontaneously flowing cerebrospinal fluid, the study solution of hyperbaric 2% prilocaine (Tachipri® Hyperbar, Nordic Pharma) at room temperature was injected over 20s associated with sufentanil 2,5 mcg and morphine 100 mcg. A multiple orifice epidural catheter was then threaded 3 cm into the epidural space, an aspiration test was performed but no drug was injected. Immediately after the procedure, parturient laid supine with a left lateral tilt to cause uterine displacement. A bladder catheter and an O2 face mask with 6 l/min O2 were applied.

Each parturient enrolled in the study received an intrathecal dose of HP determined by the CRM and the success or failure of the block was assessed as being the primary endpoint. Off noted, the assessing anaesthesiologist remained blind to the administered dose.

For the purpose of the study, a successful block was defined as a bilateral T4 sensory level [31] obtained within 15 min after intrathecal HP dose administration with no pain experienced upon incision and until the end of surgery.

Otherwise, a failure was recorded and epidural supplementation of 5 ml bolus injections of 2% lidocaine with epinephrine 1/200.000 were administered every 5 min through the epidural catheter, in order to obtain a VAS score ≤ 3.

Hypotension was defined as a 20% decrease in systolic blood pressure (SAP) compared to baseline value, recorded before spinal anaesthesia. When occurred, titration of ephedrine 5 mg or phenylephrine 100 mcg was administered at the discretion of the attending anaesthesiologist in order to keep SAP over 90% of baseline.

The surgical technique was uniform for all patients, including uterine exteriorization.

### Blinding

To ensure proper blinding throughout the study, the same anaesthesiologist prepared the study dose according to the CRM and performed the combined spinal-epidural. Another investigator, blinded to the dose, assessed the success or failure of each intrathecal block, ensured the subsequent management of the patient and collected the data throughout the study protocol. Similarly, parturient was not aware of the dose administered.

### Measurements

Demographic variables recorded in the study were: age, weight, height, body mass index, gestational age, parity and number of previous caesarean deliveries.

Regarding the new-born, weight and Apgar scores at 1, 5 and 10 min were recorded after delivery, as well as umbilical vessels pH and methemoglobinemia measured from percutaneous umbilical cord blood samples, using arterial blood analysis.

The following surgical data were also collected: time from spinal anaesthesia to baby extraction, time from baby delivery to the end of surgery, the duration of surgery and total blood loss.

Sensory level was assessed bilaterally by loss of cold and sensation at the midclavicular line and recorded every 2,5 min after intrathecal dose administration of HP (T0) during the first 15 min. Then, every 5 min until the end of the procedure, and every hour in the Post-anesthesia care unit (PACU) until the patient declared regaining full sensitivity, signifying complete resolution of the sensory block. The time to achieve Th4 bilateral level, the maximum level obtained and the total duration of sensory block were also registered.

Bromage scale (1 = no motor block, 2 = hip blocked, 3 = hip and knee blocked; and 4 = hip, knee, and ankle blocked) was used to evaluate the motor block every 15 min after spinal anaesthesia (T0) and until the end of surgery. Patients’ follow-up continued in the PACU every hour until complete recovery of motor block was observed (Bromage score = 1) and total duration of motor blockade was recorded.

Total recovery of both motor and sensory blocks allowed discharge to the care-unit.

Pain was assessed using a 10-cm horizontal visual analogue scale (VAS; 0–10 cm; 0: no pain and 10: worst imaginable pain) at skin incision, new-born delivery, uterine exteriorization, peritoneal and skin closure; in addition, at 5-min intervals throughout surgery and at 15-min intervals during the follow-up in the PACU. Thereafter, pain was evaluated every 4 h during the first postoperative day in the care-unit.

Maternal arterial blood pressure was recorded by non-invasive measurements at baseline, at 1-min intervals after drug dose administration during the first 15 min then at 2.5-min intervals until the end of surgery and every 20 min in the post anaesthesia care unit. The necessity of using vasopressors (ephedrine or phenylephrine) when hypotension occurred as well as total administered doses were recorded. Heart rate and SpO2 were monitored continuously.

Regarding side-effects, the incidence (presence or absence) of nausea, vomiting and pruritus, were recorded at 15 min intervals from intrathecal dose administration until the end of surgery and at the same time-points as pain was assessed. During the postoperative period and until hospital discharge, all parturients were questioned and examined as well for transient neurologic symptoms (TNS), urinary retention and dizziness.

From a quality point of view, maternal satisfaction (yes or not) was assessed 1 h after surgery and in the care-unit ward.

All collected data were registered anonymously, according to institutional ethics committee policy.

### Dose allocation

To provide a valid estimation of the ED95 of 2% HP with sufentanil 2,5 mcg and morphine 100 mcg for caesarean section, the study design was based on the modified CRM [32].

It is an adaptive Bayesian method, designed to estimate the targeted percentile on the response curve among several dose levels, requiring small sample of patients of around 20–30 to reach valid conclusions. Originally designed for dose-toxicity finding in oncology trials, it was then extended to dose-failure in phase II trials, notably in anaesthesiology [24].

**Fig. 1:**
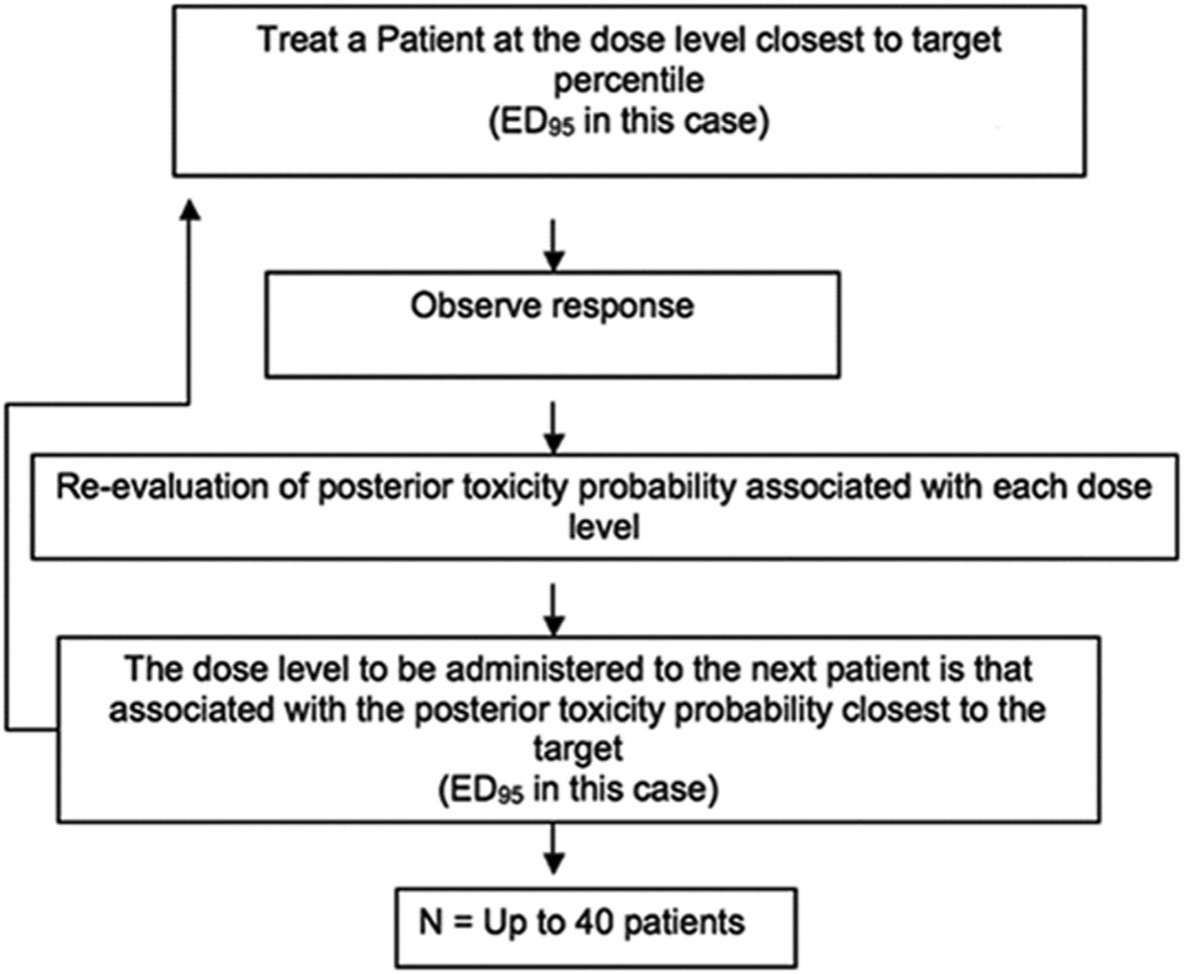
Continual Reassessment Method

We set out to recruit 40 parturients, 4 per cohort, to benefit from spinal anaesthesia with 2% HP different given doses with sufentanil. The starting dose of 45 mg was determined using a priori estimates of the ED95 based on our previous experience. Subsequent doses were allocated based on the CRM power model (Fig. 1), with the operator remaining blind to the given doses. Indeed, the results of each cohort were analysed by the statistical advisor researcher (Mr J-F. Fils) in order to propose to the clinical investigator the next dose to allocate.

### Dose-response statistical analysis

Assuming a dose-failure relationship, with higher doses being more toxic and lower doses less efficacious, we want to find the ED95; that is, the dose defined as the 5th percentile of the dose–failure relationship, which is modelled throughout a power model as follows:

$$ \mathrm{P}\left(\mathrm{Y}=1/{\mathrm{x}}_{\mathrm{i}}\right)={{\mathrm{p}}_{\mathrm{i}}}^{\uptheta}, $$

where θ is the model parameter to be estimated, considered as a random variable with exponential unit prior, x_i_ is the administrated dose to the ith patient and p_i_ (i = 1, … k) is the initial guess of failure probabilities at the *i*th dose level.

Six dose levels (= k) were chosen, specifically 30, 35, 40, 45, 50 and 55 mg, whose range was based on our previous experience. The guesstimates failure probabilities associated to the retained doses were given by clinicians as 0.5, 0.25, 0.10, 0.05, 0.02, and 0.01, a priori corresponding respectively to ED50, 75, 90, 95, 98 and 99 of HP with sufentanil.

The CRM is conducted as follows: the first cohort of four patients is administered the initial candidate of the ED95, the dose level 45 mg. Then, depending on the response observed for all patients in the cohort, Bayes theorem is applied in order to provide the actualized posterior distribution of the model parameter. Subsequently, the posterior mean estimate is computed – that is, the mean distribution after taking into account the patients recruited so far in the trial – E (θ/y), and then is used in the power model to give an updated probability of failure at each dose level. The dose allocated to the next cohort is the one with an actualized posterior response closest to the target 0.95 (95%).

CRM allows to previously incorporate stopping rules which is important for an ethically and statistically reliable approach of patients [27, 33, 34].

Our trial continued until one of the following stopping criteria was met:

1. the planned number of 40 patients was reached;
2. the estimated posterior probability of response was either too low or too high for all dose levels;
3. a reliable estimation of the ED95 was obtained, based on the predictive gains (mean and maximum) of further patients’ inclusions on the response probability and on the width of its credibility interval lower than 5%.

Collected demographic, surgical and clinical data were expressed as mean ± standard deviation or absolute number, as appropriate.

The dose-finding allocation and analysis of remaining data were performed using R software version 3.2.2 (R CRAN, Vienna, Austria).

## Results

### Demographics and surgery statistics

All 40 parturients enrolled completed the study according to the protocol and were included in the analysis. Demographics and surgery duration are presented in Table 1.

**Table 1:** Demographics and surgery characteristics

Dose	35 mg (***n*** = 4)	40 mg (***n*** = 12)	45 mg (***n*** = 20)	50 mg (n = 4)
Age (y)	29 ± 8,76	35,08 ± 5,11	32,85 ± 4,93	29 ± 5,94
Length (cm)	159,5 ± 4,93	164,42 ± 4,45	162,8 ± 6,26	157 ± 6,16
Weight (kg)	73 ± 16,02	82,17 ± 11,93	79,78 ± 11,6	84,68 ± 2,88
Gravity	3 ± 1,41	2,17 ± 1,03	3,1 ± 2,31	2 ± 1,41
Parity	1,25 ± 0,5	0,67 ± 0,49	1,3 ± 1,98	0,75 ± 0,96
Had previous CS (n)	2	5	10	1
Term (w)	38,5 ± 0,58	38,42 ± 1,08	38,40 ± 0,94	38,75 ± 1,26
Time to baby extraction (min)	12,00	8,00 ± 3,49	8,05 ± 2,95	10,25 ± 6,95
Time of surgery (min)	53,00	50,09 ± 14,02	49,32 ± 9,90	56,00 ± 15,64

Data for surgery characteristics (Time to baby extraction and time of surgery) was recorded only if success:

1 patient for the dose of 35 mg, 11 patients for 40 mg, 19 for 45 mg, 4 for 50 mg

### ED95

**Fig. 2:**
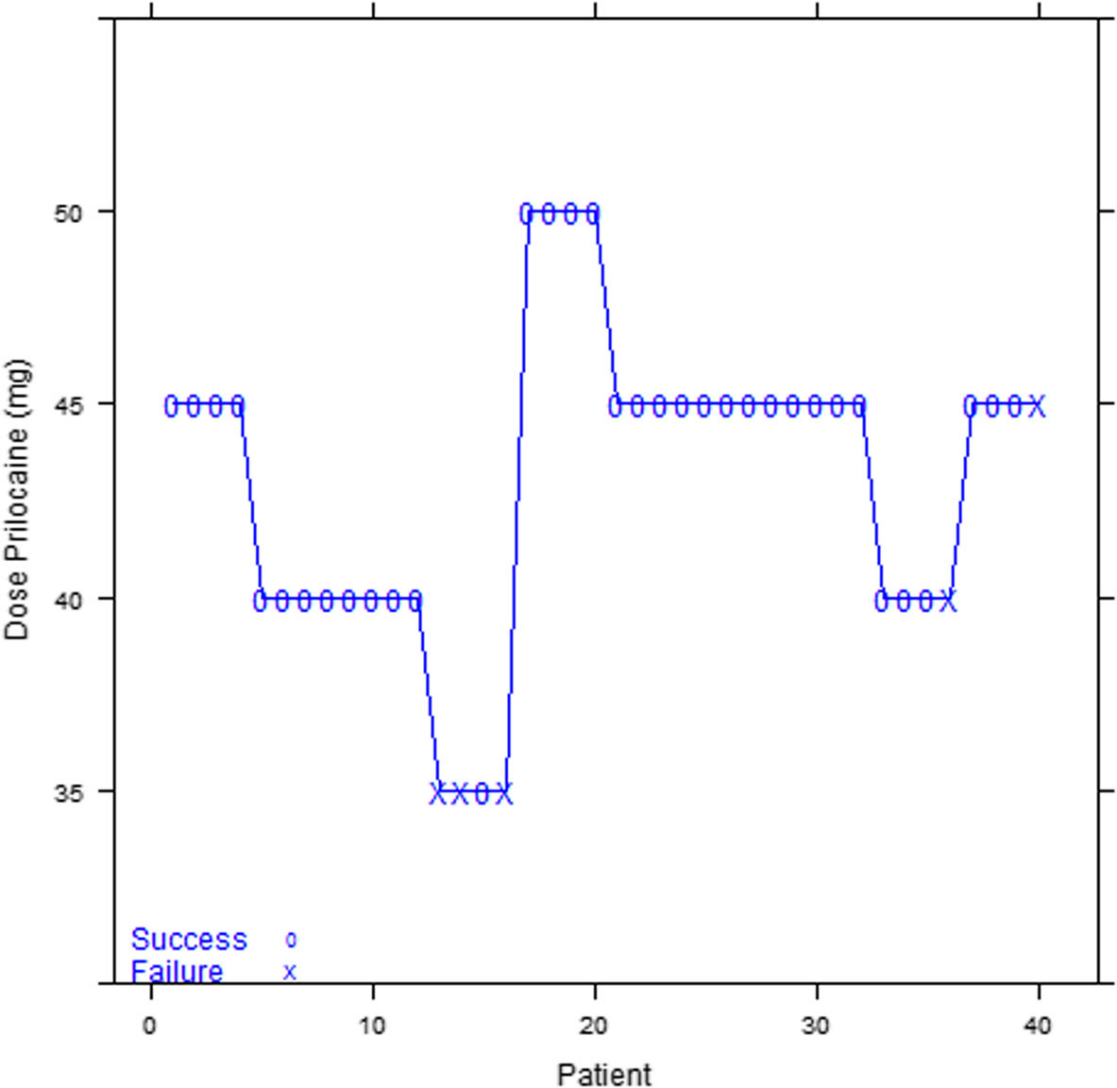
Sequence of doses

**Fig. 3:**
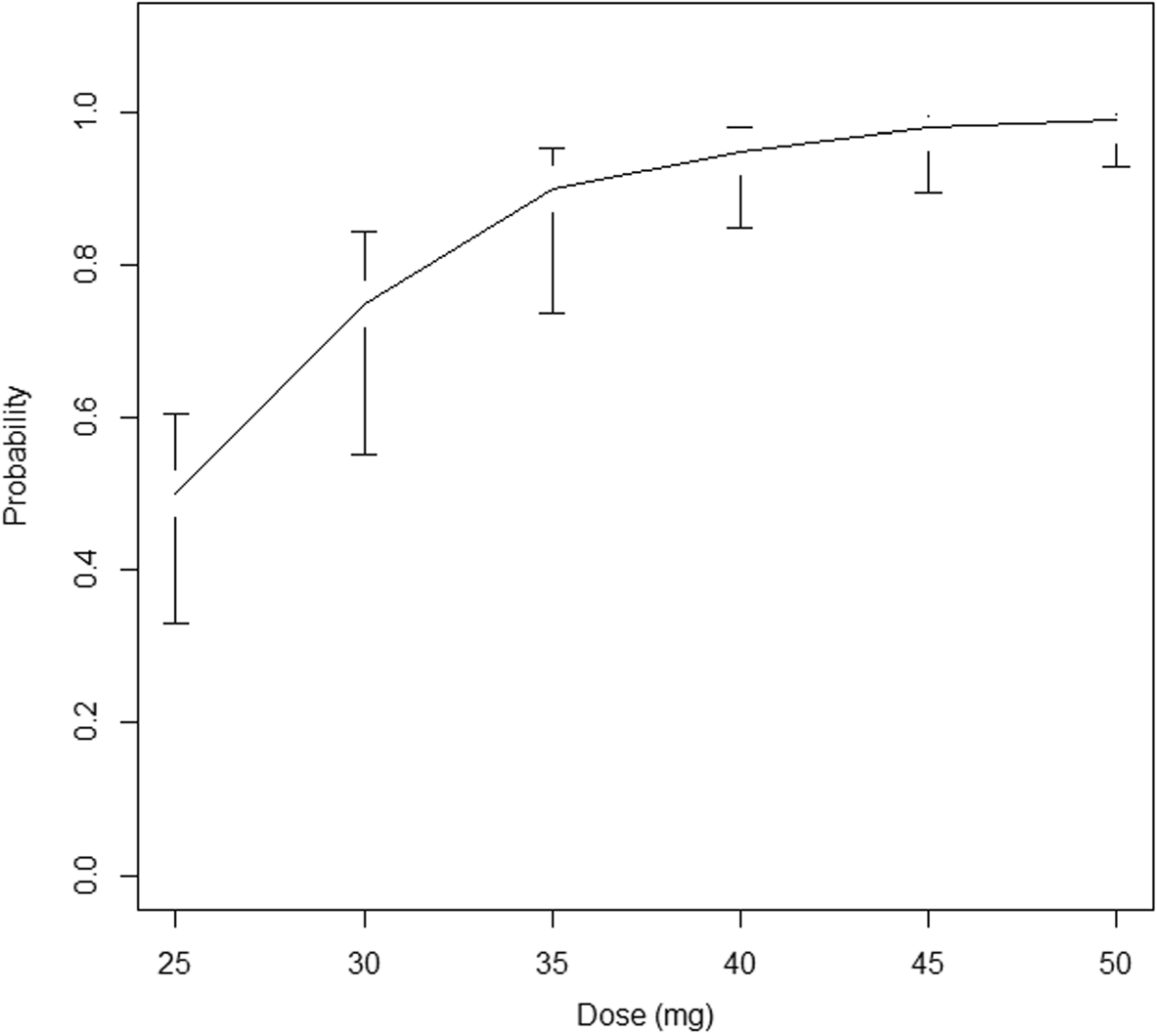
Probability of success and 95% credibilty intervals

**Fig. 4:**
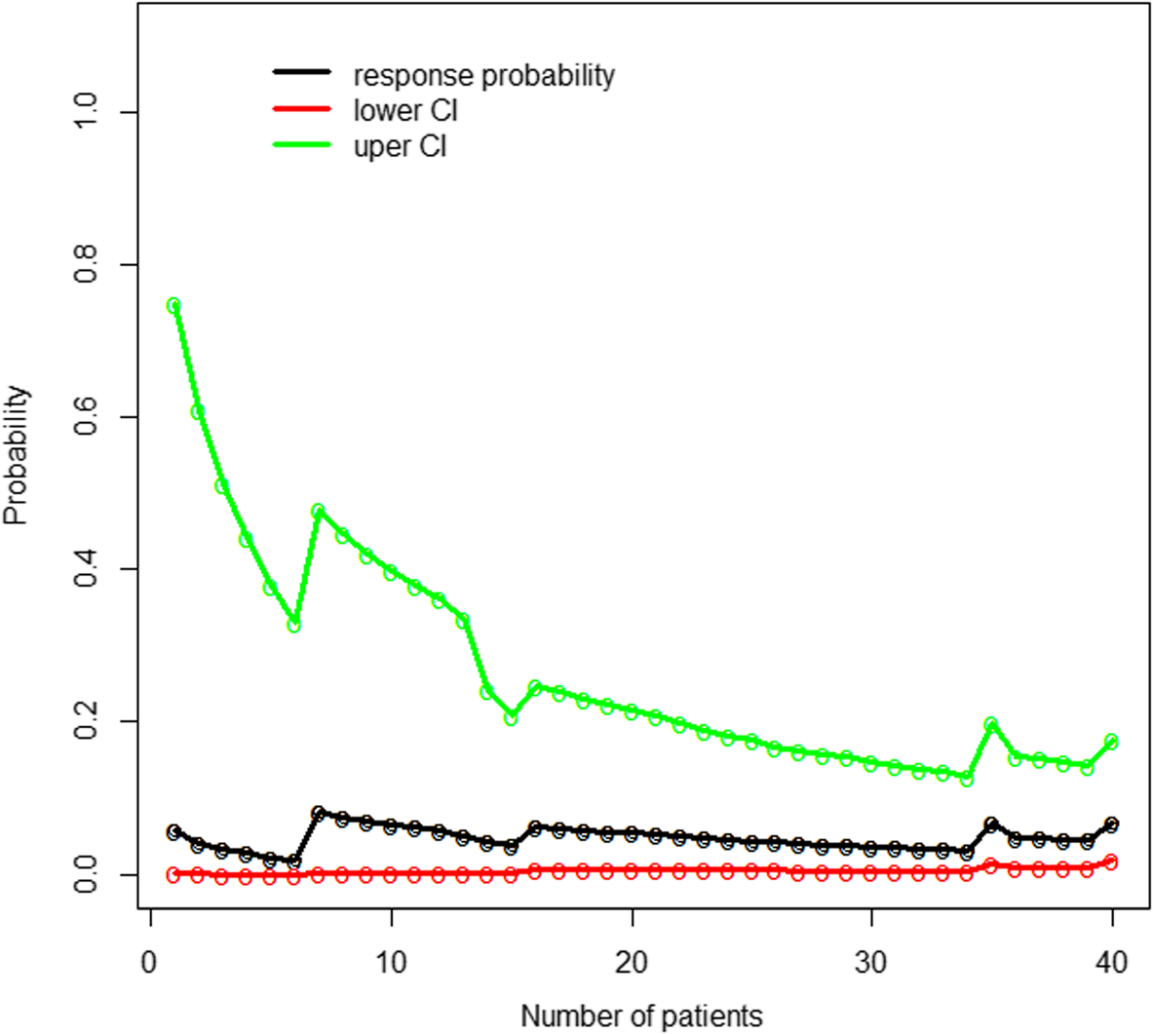
Estimated response probability and 95% credibility interval for the proposed ED95

The blocks were effective in 35 patients and ineffective in 5 patients. Figure 2 shows the sequence of administrated HP doses. Figure 3 indicates that the actualized probabilities of success associated with each of 30, 35, 40, 45, 50, and 55 mg doses are 46, 71, 87, 93, 97, and 100%, respectively. The 95% Credibility intervals were [33.10–60.50%] for dose 30, [55.25–84.40%] for dose 35, [73.70–95.43%] for dose 40, [85.00–98.19%] for dose 45, [89.66–99.47%] for dose 50 and [93.08–99.79%] for dose 55. Figure 4 depicts the estimated response probability evolution and its 95% Credibility Interval.

The doses given for each cohort varied from 35 to 50 mg of HP, according to the CRM, with a final ED95 lying between 45 and 50 mg of Prilocaine after completion of the 10 cohorts (Table 2).

**Table 2:** Evolution of ED95 after each cohort

			Prilocaine Dose, mg					
			30	35	40	45	50	55
			Working model					
			0.5	0.75	0.90	0.95	0.98	0.99
Cohort	Administered dose, mg	Clinical response	Updated Estimated Probability of Response					
1	45	S,S,S,S	0.66	0.89	**0.97**	0.99	1.00	1.00
2	40	S,S,S,S	0.73	**0.93**	0.99	0.99	1.00	1.00
3	40	S,S,S,S	0.76	**0.94**	0.99	1.00	1.00	1.00
4	35	F,F,S,F	0.48	0.73	0.88	0.93	**0.97**	0.99
5	50	S,S,S,S	0.49	0.74	0.89	**0.95**	0.98	0.99
6	45	S,S,S,S	0.51	0.76	0.91	**0.95**	0.98	0.99
7	45	S,S,S,S	0.53	0.78	0.92	**0.96**	0.99	1.00
8	45	S,S,S,S	0.54	0.79	**0.93**	0.97	0.99	0.99
9	40	S,S,S,F	0.50	0.75	0.90	**0.95**	0.98	0.99
10	45	S,S,S,F	0.46	0.71	0.87	**0.9322**	**0.9702**	0.99

In bold is the estimated posterior probability of the dose level considered to be the currently best estimate of the ED_*95*_ after the inclusion of the cohort

F = Failure, S = Success

### Secondary results of the ED95

**Fig. 5:**
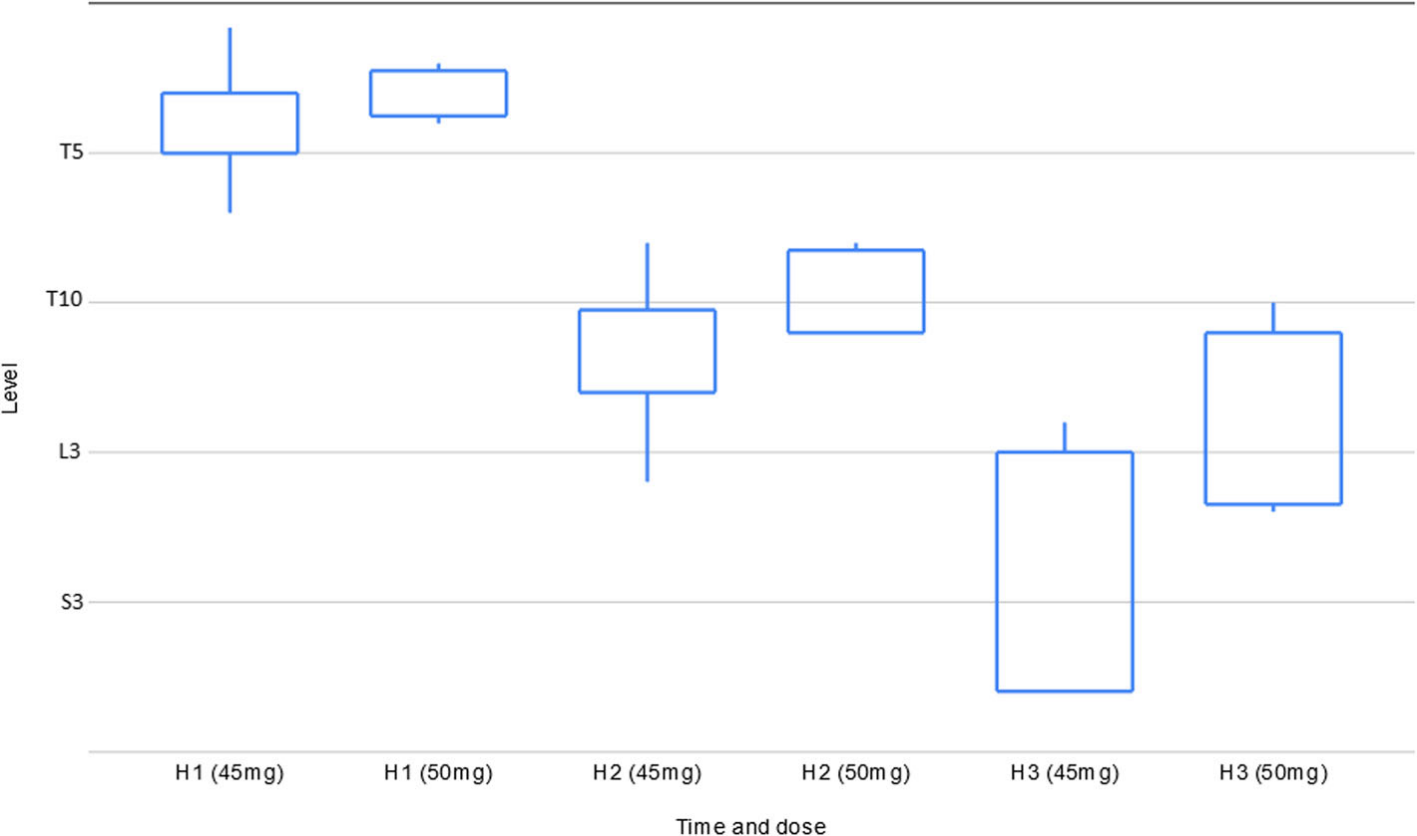
Evolution of sensitive block

**Fig. 6:**
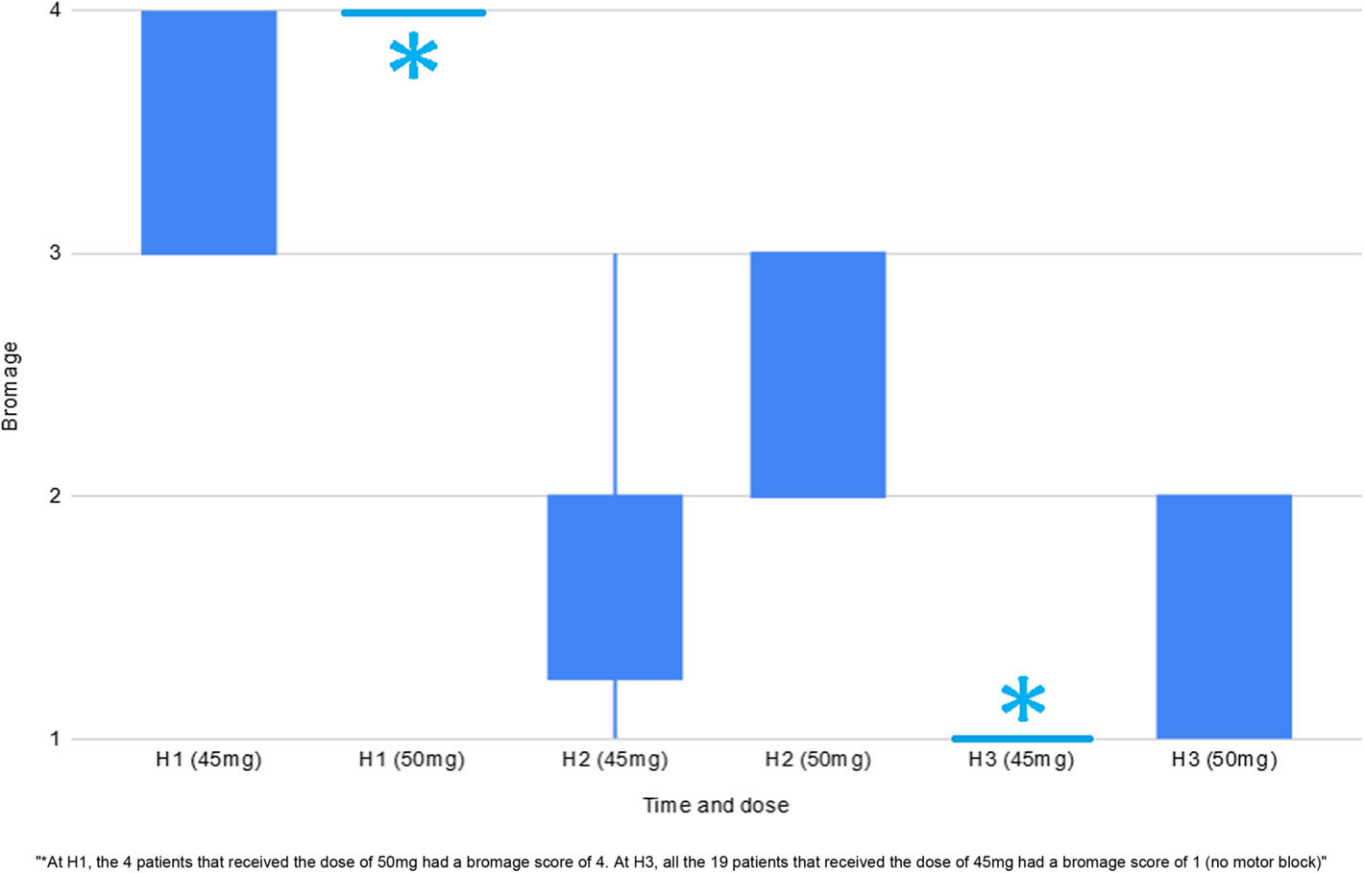
Evolution of motor block

Tables 3 to 7 and Figs. 5 and 6 present the data corresponding to the doses of 45 and 50 mg. Data was recorded only if success: 19 patients for the dose of 45 mg, 4 for 50 mg.

**Table 3:** Quality of central bloc

N	Dose (mg)	Time from injection to T4 block (min)	Sensitive block duration (h)	End of surgery sensitive block (Level)	Motor block duration (h)	End of surgery motor block (Bromage)
19	45	12,33 ± 3,52	2,31 ± 0,48	3,88 ± 1,59	2,75 ± 0,45	3,56 ± 0,51
4	50	12,50 ± 2,89	3,25 ± 0,50	3,00 ± 0,82	3,50 ± 0,58	4,00 ± 0,00

#### Sensitive and motor blocks

Mean time to T4 bilateral sensitive block was approximately 12 min, with duration of more than 2 h for the dose of 45 mg and over 3 h for the dose of 50 mg (Table 3).

Figure 5 show sensitive levels at 1, 2 and 3 h post injection for the predefined doses of 45 and 50 mg. The sensitive level at one hour post injection was over T5 for most of the patients, and decreasing rapidly each hour afterward. At hour 3, the sensitive block for the dose of 50 mg was at the lumbar level while for the dose of 45 mg it was sacral for most of the patients.

Figure 6 show Bromage scores at 1, 2 and 3 h post injection for the same doses. All the patients had a Bromage score of 3 or 4 at one hour post injection. The third hour, all the patients that received the dose of 45 mg were able to move freely.

#### Hemodynamics

Blood pressure was stable for both doses (Table 4).

**Table 4:** Hemodynamics

N	Dose (mg)	Sys pre (mmHg)	Sys post (mmHg)	Diast pre (mmHg)	Diast post (mmHg)	Pulse pre (bpm)	Pulse post (bpm)	Sat pre (%)	Sat post (%)
19	45	125,96 ± 16,20	111,03 ± 18,17	66,90 ± 15,00	63,03 ± 12,26	90,65 ± 17,95	85,00 ± 15,77	98,13 ± 1,32	98,30 ± 1,68
4	50	130,77 ± 20,47	128,58 ± 15,14	73,79 ± 18,94	70,42 ± 14,23	82,75 ± 10,94	75,00 ± 17,59	97,78 ± 1,22	98,29 ± 0,56

### Newborn parameters

Table 5 presents newborn parameters. Apgar scores at 1 min were at least 9 for the majority of babies and 10 after 5 min.

**Table 5:** Newborn parameters

N	Dose (mg)	Weight (g)	Apgar at 1 min	Apgar at 5 min	Apgar at 10 min	Cordal pH	MetHb of baby
19	45	3224 ± 443	9 [2–10]	10 [6–10]	10 [8–10]	7,15 ± 0,14	1,59 ± 0,53
4	50	3351 ± 923	9 [8–10]	10 [9–10]	10 [10–10]	7,23 ± 0,10	1,60

#### Adverse events

Regarding side effects, 17 of the 24 patients who received the dose of 45 or 50 mg needed vasopressors, 7 experienced dizziness, 3 had nausea and none showed TNS, neither pruritus nor urinary retention. The majority of patients were satisfied, 20 out of 23. This data is shown in Table 6.

**Table 6:** Adverse effects

N	Dose (mg)	Need for vasopressors	TNS	Nausea or vomiting	Pruritus	Urinary retention	Dizziness	Satisfaction
19	45	15	0	3	0	0	5	16
4	50	2	0	0	0	0	2	4

## Discussion

The ideal spinal anesthesia for elective cesarean section using the “optimal” local anesthetic dose should provide adequate surgical conditions throughout the procedure without consequent maternal or fetal adverse effects. It should provide a rapid onset of sensory and motor blocks (also interesting in a semi-emergency context) and rapid predictable regression of motor block permitting early rehabilitation, while ensuring sufficient postoperative analgesia. These qualities, together with a low incidence of adverse effects, are undoubtedly the requirements for any anesthetist in day practice.

The primary aim of the current study was to determine the ED95 of 2% intrathecal hyperbaric prilocaine, combined with sufentanil 2,5 μg and morphine 100 μg, for elective cesarean section. Using the continual reassessment method, we estimated the ED95 for successful anesthesia was between 45 and 50 mg, with most observed success with the 45 mg dosage.

The definition of a successful block differs widely amongst dose-finding studies having investigated the potency of intrathecal local anesthetics for cesarean section [1, 10, 35–37].

In this study, we defined as “success” the combination of a bilateral T4 attained sensory level obtained within 15 min after intrathecal HP dose administration with no pain experienced upon incision and until the end of surgery. We did this choice for the following reasons.

Regarding the sensory level required for CS, we aligned our practice with the current recommendations suggesting a T4/T5 dermatome, rather than a bilateral T6 adopted by previous studies [1, 35].

We also considered that a 15 min delay to attain the sensory level was more appropriate than the 10 min previously reported, in order to avoid early failures due rather to the spread than the dose itself [1]. In addition, to our knowledge, since no study on intrathecal HP has reported before the time to T4 dermatome, we believed that 15 min delay was consistent with the results concluded on bupivacaine for CS, varying between 4 and 12 min [4, 37, 38].

Overall, surgical anesthesia was effective in 35 of 40 patients (87.50%), for the predefined assessed doses, which can be consider as a high success rate comparing with reported results on other local anesthetics [10, 39].

Interestingly, our results provide evidence that a dose of HP between 45 and 50 mg is sufficient to ensure surgical anesthesia to a T4 sensory level, which is in fact lower comparing to the doses reported by previous dose-finding studies [12]. We believe that the adjuvant sufentanil may contribute in reducing the dosage of prilocaine in our study. It is well acknowledged that opioids enhance the quality of anesthesia provided by local anesthetics for caesarean delivery [9, 10, 40].

In regard to secondary results, studies investigating local anesthetics for CS, differ widely in their methodology, including the drugs, doses and methods by which the characteristics of blocks are assessed this hampering correct comparability [11].

In this study, time to attain T4 level was comparable to the one reported for levobupivacaine (the levorotatory enantiomer of bupivacaine) but longer comparing to the long-lasting hyperbaric bupivacaine [4, 10]. The duration of motor block was however shorter as expected because of the intermediate potency of HP, consistent with the short duration of surgery in our tertiary center. Importantly, no adverse hemodynamic effects were recorded in our study population, thus suggesting that prilocaine may offer an interesting perspective to the current dilemma for anesthetists “dense-better anesthesia is associated with a higher incidence and severity of hypotension” [8]. In addition, no side-effects were observed in babies and no TNS was shown, while the majority of patients were globally satisfied by the whole procedure.

Comparability with other local anesthetics being beyond of the scope of the study, we are convinced that it will be of great interest to conduct prospective randomized studies to compare HP to other established drugs in this field. Such studies should be based on equipotent doses, which were concluded for bupivacaine to range between 11 and 13 mg [1, 35] and for ropivacaine, when used alone, close to 26 mg [41]. Whereas efficient, such dosages elicit hypotension, thereby carrying a high risk for mother and fetus [6, 7].

Several trials have reported the applicability of HP, since 2005, for short surgical procedures under spinal anesthesia. However, its use has never been reported in obstetrical anesthesia yet. Today’s policies appeal for a generalization of enhanced recovery procedures. Hyperbaric bupivacaine, despite its advantage of reliable good quality block, presents side effects that are a barrier to this enhanced recovery objective. Also, its ED95 has only been calculated from the ED50.

In fact, the most used statistical method in anesthesiology for determination of a drug’s ED95 is the Up-And-Down method (UDM). The principle is that each administrated dose is determined by the success or failure of the previous one. If it was a success, next dose would be inferior, but in case of failure, the next one would be superior, aiming to the ED50. ED95 is then calculated from the dose/response curve. The major advantage is that small groups of patients are sufficient, but the estimation of ED95 from ED50 lacks of precision.

Another statistical design, the “3 + 3” method, is based on the same principle but uses cohorts of 3 patients for each dose, which give more precise information for every single dose. His disadvantage is the need to start with a low dose, which means treating patients with inefficient doses until the efficacy range is reached. Moreover, it does not provide any accurate estimate of the response rate, based at most from 6 patients.

In this study, we used the CRM, working on Bayesian inference. This statistical approach exists since the XVIIIth century, but is used in dose estimation since 1990. It is still poorly used in clinical research because unknown and complex, needing the active participation of a biostatistician to help the clinician.

Citing Prof. H. Motulsky, Bayesian approach “allows combining objective results with previous clinical intuition to calculate the probability of a patient being sick”.

For a dose/response clinical study, the clinician will use every a priori available information and complete data a posteriori with further results to establish conclusions.

The use of CRM in this study showed several advantages over UDM: not aiming at ED50 is the main one. Aiming directly at ED95 leads to treat patients with efficient doses earlier, which is ethically important. UDM uses logistic regression to estimate ED95, where CRM uses a one parameter model to directly estimate ED95, more precisely. It uses all information available to give each patient the lowest efficient dose.

It’s liability is better as it uses the information of every cohort to estimate the ED95, where UDM uses only the previous patient result.

O’Quigley, which used CRM for the first time in 1990 for phase I clinical trials in cancer, concludes superiority of CRM over UDM because it “learns” from information obtained at earlier points in the study. Consequently, it is less likely to treat patients at toxic doses, and more likely to treat patients at effective doses [25, 42]. Notably, it has been extended to phase II dose-finding clinical trials to estimate the minimal effective dose of a new drug [34].

CRM avoids treating patients with toxic doses by setting limitation rules restraining the trespassing of superior and inferior doses. It also allows a more rapid variation of dose than UDM. Those rules have to be adapted with each study design. In our, we followed advice from statisticians based on Zohar and Chevret’s model [27].

While it is true that the complexity of the model restrains its use in clinical practice, needing to work with a biostatistician, this collaboration appeared to be interesting and stimulating, with the participation of an external and different point of view. Another limitation of our study may be considered the choice of the sensory block assessment, however, consensus on the best method is warranted [31].

In conclusion, the ED95 of intrathecal hyperbaric prilocaine with sufentanil 2.5 μg and morphine 100 μg for elective cesarean delivery was found to be between 45 and 50 mg. Taking in consideration the good quality provided sensitive block combined with early rehabilitation, hemodynamic tolerance and good babies’ outcome, hyperbaric prilocaine may be an interesting alternative to other long-lasting local anesthetics in the context of scheduled cesarean delivery.

## Data Availability

Results of the current study are published at https://clinicaltrials.gov/ct2/show/results/NCT03036384. The complete datasets used and analyzed are available from the corresponding author upon request. They are deposited in the electronic database of the Anesthesiology department, University Hospital Saint Pierre. The CRM estimation data are deposited in the electronic database of our biostatistician.

## Abbreviations

ASA: American Society of Anesthesiologists
CRM: Continual reassessment method
CS: Cesarean section
CSE: Combined spinal-epidural
ED50: 50% effective dose
ED95: 95% effective dose
HP: Hyperbaric prilocaine
PACU: Post-anesthesia care unit
SAP: Systolic blood pressure
SpO2: Pulse Oximetry
TNS: Transient neurologic symptoms
UDM: Up-and-down method
VAS: Visual analogue scale
